# “Dissecting the role of T cell exhaustion in cancer progression: a multifaceted approach“

**DOI:** 10.3389/fimmu.2025.1646292

**Published:** 2025-12-03

**Authors:** Xinyang Li, Shiwen Song

**Affiliations:** 1 Department of General and Vascular Surgery, Shengjing Hospital of China Medical University, Shenyang, Liaoning, China; 2 Department of Thoracic Surgery, The First Affiliated Hospital of China Medical University, Shenyang, Liaoning, China

**Keywords:** T cell exhaustion, TME, immune escape, epigenetic reprogramming, clinical translation, immunotherapy

## Abstract

This article thoroughly explores the crucial role of T cell exhaustion in the process of tumor immune escape, comprehensively explaining its key characteristics, such as dynamic plasticity, heterogeneity, and epigenetic reprogramming. The article first elaborates on the complex interaction between immune surveillance and tumor escape, and then clarifies the core position of T cells in anti-tumor immunity and the evolution of the “exhaustion” concept, covering various research fields from chronic infections to the tumor microenvironment (TME). It provides a detailed analysis of the origin, differentiation pathways, and dynamic plasticity of exhausted T cells, revealing the possibility of functional recovery under specific conditions. At the same time, the article analyzes the profound influence of various factors in the TME (such as metabolic stress, immune suppression networks, and stromal interaction interfaces) on the process of T cell exhaustion. It conducts in-depth research on the molecular characteristics of exhausted T cells (including surface marker characteristics, transcriptional regulatory networks, and metabolic reprogramming characteristics), providing potential therapeutic targets for precision medicine. In the clinical translation aspect, this study clarifies the cutting-edge exploration achievements of diagnostic biomarkers, such as the exhausted subtypes defined by single-cell multi-omics technology, the prognostic value of TCR clonal dynamics, and the innovation of treatment strategies, including the “re-mobilization window” theory in PD-1 blockade, the synergistic effect of epigenetic drugs, the temporal and spatial selection in metabolic intervention, and the application of engineered cell therapies. This study systematically integrates the latest progress in the field of T cell exhaustion, providing comprehensive and profound theoretical support and innovative ideas for addressing challenges in tumor immunotherapy.

## Graphical Abstract

**Figure:**
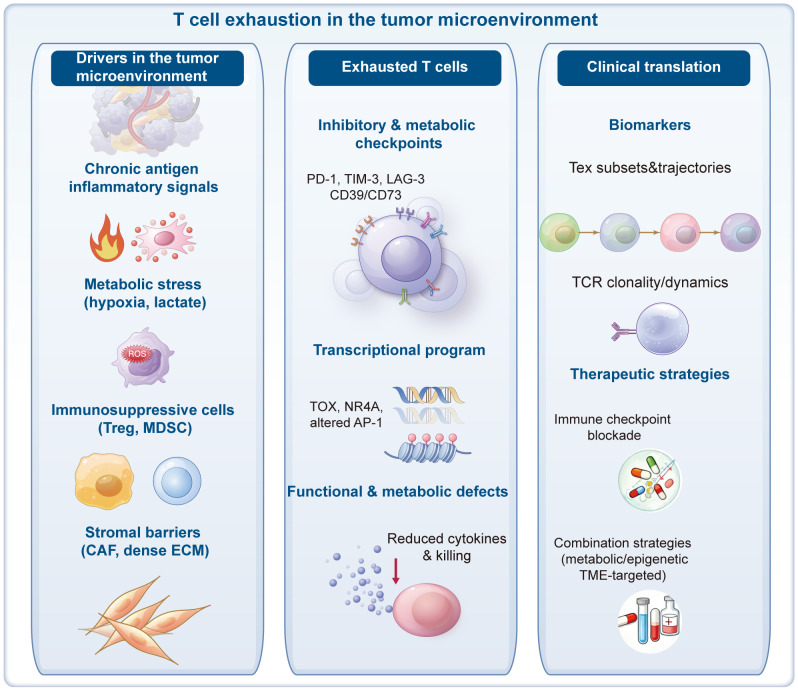

## 1 Introduction

### 1.1 The game between immune surveillance and tumor escape

In the intricate physiology of humans, the immune surveillance mechanism functions as a loyal sentinel, constantly vigilant against the emergence of abnormal cells, especially during the early stages of tumorigenesis (1–3). The immune system, with its formidable recognition capabilities, precisely identifies cells that undergo genetic mutations and deviate from normal growth trajectories, with T cells playing a pivotal role as executors in this process (4–6). These T cells equipped with T-cell receptors (TCRs) on their surfaces (7, 8), act like specialized “scanners.” They specifically recognize abnormal antigen peptides presented by major histocompatibility complex (MHC) molecules on tumor cells (9). Upon locking onto their targets, T cells swiftly activate and initiate a complex series of immune response programs. For example, naive T cells are instantaneously activated and break their resting state upon receiving tumor antigen signals from antigen-presenting cells (such as dendritic cells), along with essential co-stimulatory signals (10, 11). These activated T cells, akin to soldiers receiving combat orders, begin to proliferate extensively, with their numbers surging exponentially in the short term, forming a massive army of immune cells. These proliferating T cells differentiate into effector T cells, with cytotoxic T cells (CD8+ T cells) directly targeting the location of tumor cells. By secreting cytotoxic molecules such as perforin and granzyme B, they precisely punch holes in the tumor cell membranes, inducing apoptosis (12–14). Simultaneously, they secrete cytokines such as interferon-γ (IFN-γ) and tumor necrosis factor-α (TNF-α), which not only directly inhibit tumor cell growth but also recruit and activate other immune cells, such as macrophages and natural killer (NK) cells, to collaborate in forming a comprehensive immune attack network, striving to annihilate tumor cells in one fell swoop (15, 16).

However, tumor cells are not helpless targets. Over the course of long-term evolution, they have gradually learned multiple “tricks” to escape immune surveillance. Some tumor cells downregulate the expression of MHC molecules, making it difficult for T cells to recognize the antigen peptide-MHC complexes on their surfaces, akin to “camouflaging” their characteristics to evade the “scanner” of T cells (17, 18). Some tumor cells also overexpress programmed death-ligand 1 (PD-L1). The binding of PD-L1 to its receptor PD-1 on T cells transmits strong inhibitory signals. This interaction forces T cells into a functionally suppressed state, akin to pressing a “pause button” that significantly reduces their attack capabilities (19, 20).

Even more cunningly, tumor cells secrete various immunosuppressive cytokines, such as transforming growth factor-β (TGF-β) and interleukin-10 (IL-10) (21, 22), creating an immunosuppressive atmosphere in the tumor microenvironment (TME). These cytokines not only directly inhibit T-cell activity, limiting their proliferation and cytotoxic function but also attract and activate regulatory T cells (Tregs) and myeloid-derived suppressor cells (MDSCs) (23–26). Tregs and MDSCs act as “accomplices” of tumor cells, further suppressing the function of effector T cells through various mechanisms, such as consuming nutrients essential for T-cell survival or secreting more immunosuppressive molecules, thereby plunging the entire TME into a stalemate favorable for tumor growth but unfavorable for immune attacks.

#### 1.1.1 The central role of T cells in antitumor immunity

As a key force in the adaptive immune system, T cells occupy a central position in antitumor immunity, their role spanning all stages of tumorigenesis and development (27, 28). In the initial stages of tumorigenesis, T cells, with their highly specific antigen recognition ability, can precisely identify those cells that have just become abnormal and promptly eliminate them, akin to extinguishing a fire as soon as it starts smoking, preventing the disaster from escalating further (29, 30).

As tumors develop, the game between T cells and tumor cells intensifies. During this process, T cells undergo a series of complex changes. When T cells are continuously stimulated by tumor antigens and influenced by immunosuppressive factors in the TME, their functions gradually deteriorate, ultimately entering a state known as “exhaustion” (31, 32). Exhausted T cells undergo significant changes on their surfaces, such as high expression of a series of inhibitory receptors, including PD-1, cytotoxic T-lymphocyte-associated protein 4 (CTLA-4), lymphocyte-activation gene 3 (LAG-3), etc. These receptors serve as “chains” on T cells. When they bind to corresponding ligands in tumor cells or the TME, they continuously transmit inhibitory signals into T cells, reducing their proliferative capacity, cytokine secretion, and cytotoxic function (33–35).

Exhausted T cells gradually lose their combat effectiveness against tumor cells in the TME, and their original immune surveillance function is greatly weakened (36, 37). This allows tumor cells to grow and spread freely in a relatively relaxed immune environment, accelerating the deterioration of the condition. In a sense, the emergence of T-cell exhaustion is an important hallmark of tumor immune escape and one of the key factors leading to poor tumor treatment outcomes (38, 39).

#### 1.1.2 The evolution of the exhaustion concept: from chronic infection to the tumor microenvironment

The concept of exhaustion initially emerged in studies of chronic infections. During chronic viral infections, such as human immunodeficiency virus (HIV) infection and hepatitis C virus infection, the virus persists in the body, continuously stimulating the immune system (40, 41). T cells, in response to these chronic viral infections, gradually exhibit a functionally abnormal state. Unlike T cells in acute infections that efficiently clear viruses, in chronic infections, although T cells can continuously recognize viral antigens, their proliferation, cytokine production, and cytotoxic functions gradually decline. Studies have found that these exhausted T cells highly express inhibitory receptors, such as PD-1, and their gene expression profiles also undergo significant changes, exhibiting a unique transcriptional signature (41, 42). This discovery provides a new perspective for understanding the immune response under long-term antigen stimulation and lays the foundation for subsequent research on T-cell exhaustion in the TME.

When researchers turned their attention to the TME, they found that T cells there also exhibited a similar state of exhaustion (42, 43). The TME is a complex and highly heterogeneous ecosystem composed of tumor cells, immune cells, fibroblasts, vascular endothelial cells, various cytokines, metabolites, and other components. In such an environment, T cells are influenced by multiple factors. Tumor cells continuously release tumor antigens, keeping T cells in a state of continuous antigen stimulation. Meanwhile, immunosuppressive cells (such as Tregs and MDSCs) and immunosuppressive cytokines (such as TGF-β and IL-10) abundantly exist in the TME, further exacerbating the process of T-cell exhaustion. Compared with exhausted T cells in chronic infections, those in the TME have their unique characteristics. On the one hand, the complexity of the TME makes the regulatory mechanisms of T-cell exhaustion more diverse, involving not only the expression of inhibitory receptors but also multiple factors such as changes in cell metabolism and epigenetic reprogramming (42, 44). On the other hand, T-cell exhaustion in the TME is closely related to tumor progression and patient prognosis, playing a more prominent role in tumor immune escape.

Over time, as research progresses, we have gained a clearer understanding of the evolutionary history of T-cell exhaustion. From the initial discovery of exhaustion phenomena in chronic infections to the in-depth analysis of its molecular mechanisms, intercellular interactions, and effects on immunotherapy in the TME, this concept has continued to expand and deepen. We gradually recognize that T-cell exhaustion is not a single, static process but a dynamic, multi-stage evolutionary process finely regulated by various factors inside and outside the body. A deep understanding of this process provides a critical theoretical basis for the development of targeted immunotherapy strategies and brings new hope for overcoming the medical challenge of tumors.

### 1.2 States of exhaustion

#### 1.2.1 Origins and differentiation of exhausted T cells

**Figure 1:**
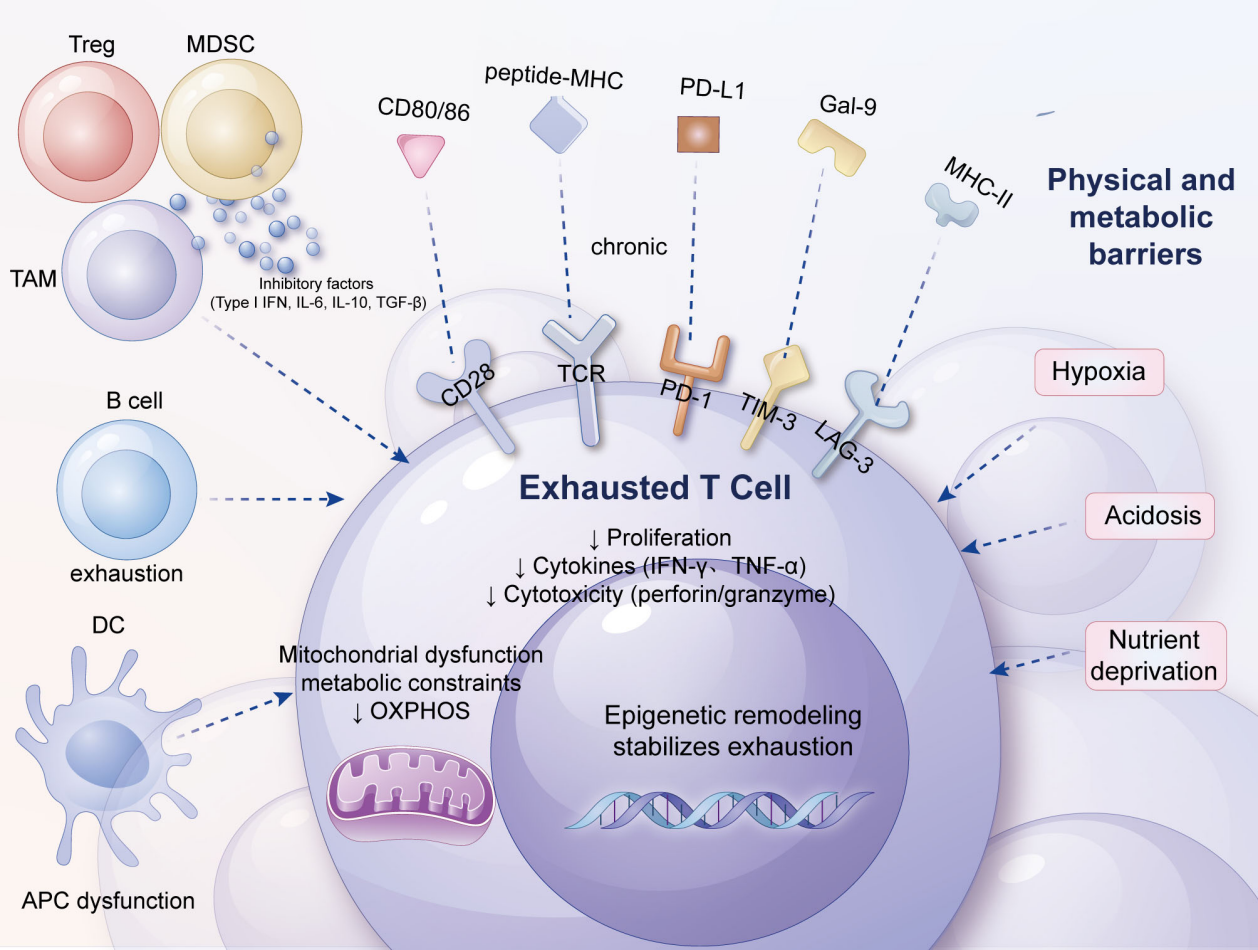
Factors contributing to T-cell exhaustion. This figure depicts the interconnected factors driving exhaustion in tumor-infiltrating T cells. From the outermost to innermost layers, chronic antigen stimulation, imbalanced co-stimulatory/co-inhibitory signaling, and a complex network of soluble cytokines (e.g., type I IFNs, IL-6, IL-10, TGF-β) interact with physical and metabolic barriers, including hypoxia, acidosis, and nutrient depletion. Dysfunctional dendritic cells, abundant regulatory T cells (Tregs), and myeloid-derived suppressor cells (MDSCs) further amplify local immunosuppression, while stromal fibroblasts and abnormal vasculature create a hostile niche that limits T-cell trafficking and survival. Together, these extracellular cues converge on intracellular signaling, metabolic rewiring, and epigenetic reprogramming, locking T cells into a stable exhausted phenotype and fostering tumor immune escape.

During acute infections, naive T cells differentiate into effector T cells and memory T cells after activation. Effector T cells undergo apoptosis shortly after clearing the pathogen. However, under long-term antigen stimulation (such as in chronic inflammation or tumors), T cells that continuously battle antigens gradually enter a state of exhaustion, leading to impaired function. As the degree of T-cell exhaustion increases, pathogens or tumor cells proliferate and spread, causing severe damage to the body (45) (**Figure 1**).

Long-term antigen stimulation is only one of the factors leading to T-cell exhaustion. Overall, these factors can be divided into the following three categories. Firstly, intercellular communication signals, such as continuous antigen stimulation and overexpression of inhibitory receptors. Secondly, the role of soluble cytokines, such as type I IFN, IL-10, and TGF-β, which can inhibit T-cell function under specific conditions (46–48). Thirdly, the influence of tissue and the microenvironment, involving changes in the expression levels of chemokine receptors, adhesion molecules, and nutrient receptors, which collectively shape the microenvironment in which T cells reside and, consequently, affect their functional state.

Apart from the aforementioned factors, other types of immune cells and stromal cells may also promote T-cell exhaustion through the aforementioned mechanisms. For example, in the TME, Tregs can secrete immunosuppressive factors such as IL-10 and TGF-β, directly inhibiting the function of effector T cells (49). Tumor-associated macrophages (TAMs) further propel the exhaustion process of T cells through multiple mechanisms, including the production of IL-10, adenosine, and reactive oxygen species (ROS), as well as the expression of PD-L1 (50). Additionally, TAMs, dendritic cells (DCs), and MDSCs can deprive T cells of essential nutrients (such as arginine and tryptophan), further weakening T-cell activity and effector function, pushing them into a state of profound exhaustion (51, 52).

#### 1.2.2 Dynamic plasticity of exhaustion states

Delving into the dynamic plasticity of T-cell exhaustion states is one of the core issues in the field of tumor immunology research. In the TME, T cells do not remain in a fixed state of exhaustion but exhibit a certain degree of dynamic plasticity, meaning that their functional states can adjust according to changes in the microenvironment and the stimuli received (53, 54). This dynamic plasticity has important clinical implications in tumor immunotherapy.

On the one hand, plasticity is manifested in the partial or even complete restoration of function in exhausted T cells under specific conditions. For instance, the application of immune checkpoint inhibitors (such as anti-PD-1 and anti-CTLA-4 antibodies) provides compelling evidence for the functional recovery of exhausted T cells (53, 54). When these drugs block the binding of inhibitory receptors on the surface of T cells to their ligands, the inhibited T-cell signaling pathways are reactivated, enabling some exhausted T cells to regain proliferative capacity, increase cytokine secretion, and enhance their killing effect on tumor cells (42, 55). This indicates that the internal signaling transduction mechanisms of exhausted T cells are not completely ineffective but rather in a reversible state of inhibition. By lifting key inhibitory signals, they still have the opportunity to “reignite” and re-engage in antitumor immune responses.

On the other hand, the dynamic plasticity of T-cell exhaustion is also influenced by the dynamic factors within the complex TME. For instance, fluctuations in oxygen content, nutrient supply, and immune cell composition in the TME can affect the functional state of exhausted T cells (42, 43). Under oxygen-sufficient conditions, the metabolic pathways of T cells change, potentially enhancing their energy production capabilities, thus supporting their functional recovery. In contrast, when nutrients are scarce, the metabolic activities of T cells are restricted, and the state of exhaustion may further intensify. Furthermore, the interactions between immune cells in the TME are also in dynamic flux (42, 54). For example, as immunotherapy progresses, the number and activity of Tregs and MDSCs in the TME may change, which will correspondingly affect the immunosuppressive environment in which exhausted T cells reside, thereby influencing their plasticity.

However, the regulatory mechanisms underlying the dynamic plasticity of T-cell exhaustion have not yet been fully elucidated. In-depth investigation of this issue requires the integrated use of various technical means, such as single-cell sequencing technology, real-time imaging technology, and high-throughput screening technology. Single-cell sequencing enables precise analysis of the dynamic changes in gene expression in individual T cells during exhaustion, revealing molecular characteristics at different stages. Real-time imaging technology allows direct observation of the behavioral changes of T cells in the TME and their interactions with other cells. High-throughput screening technology facilitates the rapid identification of key genes and signaling pathways that affect the plasticity of T-cell exhaustion. The application of these technological means will provide strong support for our comprehensive understanding of the dynamic plasticity of T-cell exhaustion and lay the foundation for the development of more precise and effective immunotherapy strategies.

#### 1.2.3 Heterogeneity boundary between exhaustion and dysfunction

The heterogeneity boundary between T-cell exhaustion and dysfunction is another challenging scientific issue. In the TME, T-cell populations exhibit high heterogeneity, with significant differences in cell surface molecule expression, cytokine secretion patterns, cytotoxic functions, and responses to immunotherapy among different T cells (44, 56). This heterogeneity makes it difficult to define the state of T-cell exhaustion using a single indicator or feature, posing challenges for accurately assessing T-cell functional status and predicting immunotherapy outcomes (57).

**Figure 2:**
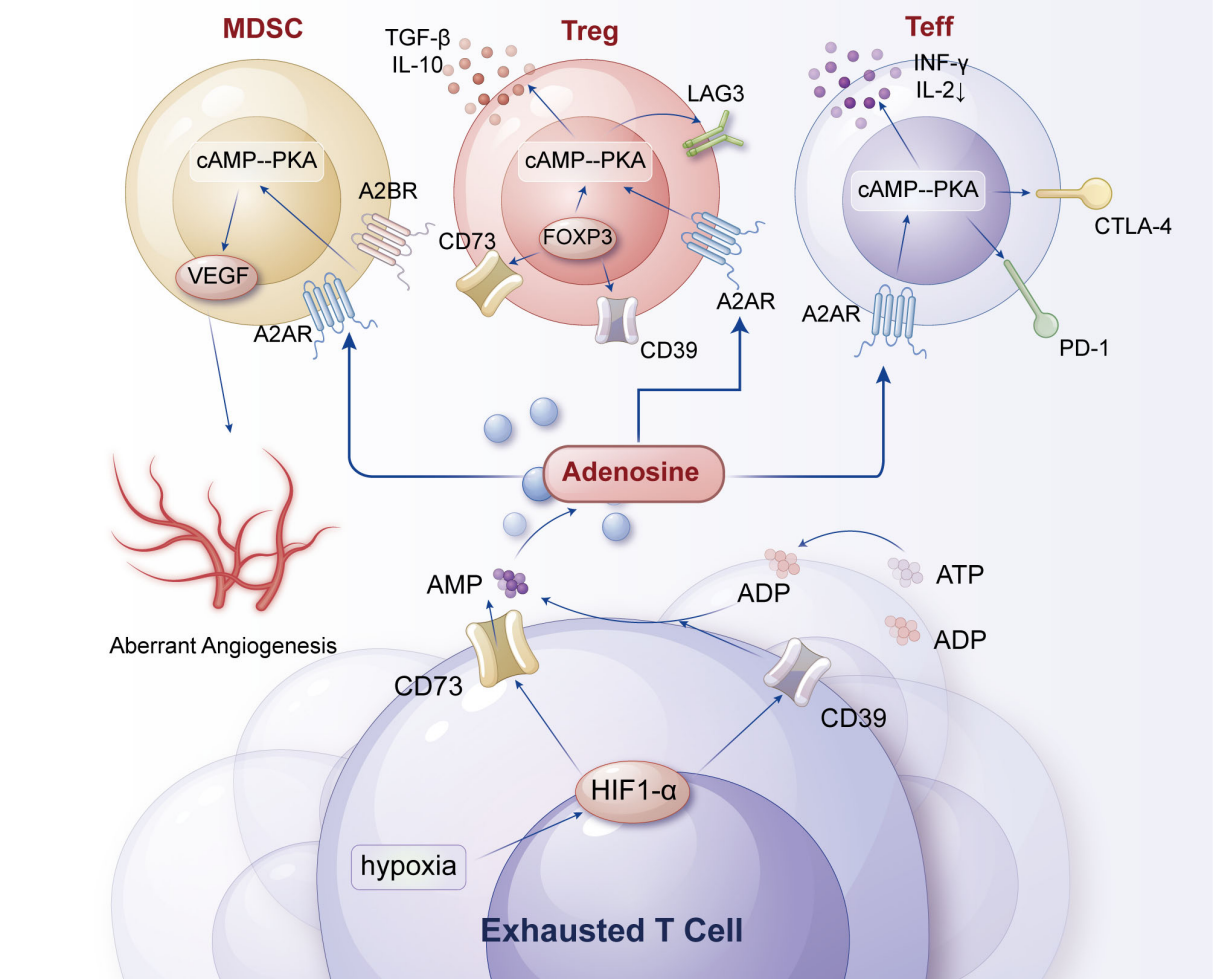
Developmental pathway of exhausted T cells. During acute immune responses, naïve T cells differentiate into short-lived effector cells and long-lived memory populations once the pathogen or antigen is cleared. In contrast, persistent antigen exposure and chronic suppressive signals within the TME divert differentiation toward a unidirectional exhaustion pathway. Early in this process, progenitor-exhausted cells (T_ex-prog; TCF-1^+) retain partial proliferative and functional capacity and can be reinvigorated by immune checkpoint blockade. With continued stimulation, they transition through intermediate states into terminally exhausted cells (T_ex-term; TCF-1^−), characterized by high co-expression of multiple inhibitory receptors, profound metabolic defects, and near-complete loss of effector function. The figure also highlights potential “intervention windows” where therapeutic strategies may redirect differentiation back toward functional effector or memory fates.

Studies have shown that even within the tumor tissues of the same patient, T cells in different regions may be in varying degrees of exhaustion (42, 56). Within the tumor immune microenvironment, the exhausted T-cell population exhibits significant heterogeneity, which can be subdivided into progenitor exhausted T cells (Texprog), intermediate exhausted T cells (Texint), and terminally exhausted T cells (Tterm) based on their developmental progression (58) (**Figure 2**). Texprog are at the initial stage of the exhaustion process, with relatively low expression of inhibitory receptors on their cell surfaces and retention of a certain degree of proliferative potential and cytokine secretion ability. Texint are in a transitional stage of the exhaustion process, with increased expression levels of inhibitory receptors compared to Texprog, decreased cytokine secretion ability, and limited proliferative capacity. Tterm represent the terminal stage of the exhaustion process, characterized by high expression of inhibitory receptors on their cell surfaces, nearly complete loss of cytokine secretion ability, and extremely low proliferative capacity, existing in a state of profound dysfunction (**Table 1**).

**Table 1:** Transcription factors and molecule expression profiles in different exhausted T cell subsets.

Transcription factor	Effector T cell	Progenitor exhausted T cell	Terminally exhausted T cell
TCF-1	High	High	Low
Ratio of T-bet and EOMES	More T-bet	More T-bet	More EOMES
BATF	Complexed with IRF4 promotes effector function	High	High
NFAT	Heterodimer with AP-1	Unclear	NFAT homodimer
PRDM1	High	Low	High
NR4A	Low	High	High
TOX	Low	High	High

However, in contrast to effector T cells, the developmental trajectory of exhausted T cells more closely resembles a “one-way path” (37). After being activated during acute infections, effector T cells efficiently eliminate pathogens within a short period and subsequently undergo apoptosis, with a minority differentiating into memory T cells upon completion of the immune response. Conversely, in the context of chronic infections or the TME, the dual impact of persistent antigen stimulation and an immunosuppressive microenvironment gradually propels T cells into a state of exhaustion. During their development, exhausted T cells undergo significant alterations in their gene expression profiles, with a gradual downregulation of genes associated with cell proliferation, cytokine production, and cytotoxicity, and an upregulation of genes related to inhibitory receptors and immunosuppression. This reshaping of gene expression leads to the gradual loss of normal immune effector functions in exhausted T cells, while continually reinforcing their immunosuppressive phenotype. Consequently, these cells find it difficult to regain normal antitumor activity within the TME, ultimately becoming one of the pivotal factors in tumor immune escape. Some T cells may express only one or a few inhibitory receptors, with only a slight decrement in their cytokine secretion capacity; whereas others may concurrently exhibit high expression of multiple inhibitory receptors, completely losing their cytotoxic function and existing in a profound state of exhaustion. This heterogeneity may stem from microenvironmental disparities in different regions of the TME, such as oxygen gradients, cytokine concentration gradients, and uneven distributions of immune cells (31, 45). Furthermore, the inherent characteristics of T cells, including their degree of differentiation, origin, and receptor specificity, may also influence their functional status within the TME, thereby exacerbating the heterogeneity of exhaustion and dysfunction.

#### 1.2.4 Microenvironment-driven epigenetic reprogramming

The TME plays a crucial role in driving epigenetic reprogramming in T cells, a process that profoundly affects the exhaustion process and functional state of T cells (36, 59). Epigenetic reprogramming involves multiple layers, including DNA methylation, histone modifications, and chromatin remodeling, which can regulate gene expression patterns, leading to functional transformations in T cells during their long-term adaptation to the TME (60).

In terms of DNA methylation, immunosuppressive factors in the TME may alter the methylation patterns of specific regions of the T-cell genome (61, 62). For instance, promoter regions of genes related to T-cell activation and cytotoxic function may undergo hypermethylation, inhibiting the transcription of these genes and suppressing T-cell function (63, 64). Simultaneously, genes related to the expression of inhibitory receptors may exhibit increased expression levels under demethylation, further exacerbating the state of T-cell exhaustion (65, 66).

In terms of histone modifications, the TME can influence the acetylation, methylation, and phosphorylation states of histones within T cells. For example, histone deacetylases (HDACs) may be activated in the TME, leading to decreased histone acetylation levels and gene transcription inhibition (67). This makes it difficult for T cells to normally express a series of genes crucial for antitumor immunity, such as cytokine genes (e.g., IFN-γ and TNF-α) and costimulatory molecule genes, thereby weakening the immune function of T cells (68). Furthermore, the activities of certain histone methyltransferases and demethylases may change under the stimulation of the TME, affecting the expression regulation of specific genes and, consequently, the differentiation and functional states of T cells.

Chromatin remodeling is also an important aspect of microenvironment-driven epigenetic changes in T cells. Cytokines and signaling molecules in the TME can activate chromatin remodeling complexes within T cells, altering chromatin structure and thereby affecting gene accessibility (69, 70). For example, some chromatin regions that were originally in an open state and available for transcription factor binding may become tight under the influence of the TME, making it difficult for genes related to antitumor immunity to be transcriptionally activated. Conversely, chromatin regions where genes related to immunosuppression are located may become more open, promoting their expression (71).

The game between immune surveillance and tumor escape, the central role of T cells in antitumor immunity, and the evolution of the exhaustion concept collectively highlight the complexity and opportunities in the field of tumor immunology. This article aims to review the research progress on key scientific issues such as the dynamic plasticity of T-cell exhaustion states, the heterogeneity boundary between exhaustion and dysfunction, and microenvironment-driven epigenetic reprogramming. By doing so, we hope to reveal the underlying mechanisms of tumor immune escape and provide a solid theoretical foundation and innovative ideas for the development of more effective immunotherapy strategies.

#### 1.2.5 Sex-biased differences in T cell exhaustion trajectories and therapeutic responses

There are gender differences in the efficacy of immunotherapy and the incidence of autoimmune toxicity observed across different cancer types, for example, men have higher rates of objective response to PD-1/PD-L1 inhibitors in certain tumors, while women tend to experience more severe immune-related adverse events (72). In the case of women, estrogen can enhance T cell response and survival through the ERα signaling pathway, while androgens may have immunosuppressive effects (73, 74). Understanding the importance of gender differences in the design of personalized treatment strategies in the future, considering patient gender as a combination therapy or dosing regimen may be a specific population or greater benefit.

#### 1.2.6 Literature search strategy

This review synthesizes current understanding of T cell exhaustion based on a comprehensive analysis of literature retrieved from PubMed, Scopus, and Web of Science databases, covering publications from January 2000 to December 2024. Search terms included ‘T cell exhaustion,’ ‘TME,’ ‘immune checkpoint inhibitors,’ ‘epigenetic reprogramming,’ ‘metabolic reprogramming,’ and ‘CAR-T.’ Included were original research articles, clinical trials, and high-impact reviews focusing on mechanistic insights, biomarker discovery, and therapeutic innovations. Excluded were studies not peer-reviewed, non-English articles, and those not directly relevant to T cell biology in cancer.

## 2 Molecular fingerprint of exhausted T cells

### 2.1 Surface marker profile

#### 2.1.1 Synergistic regulation of classical inhibitory receptors (PD-1, TIM-3, LAG-3)

In the complex ecology of the tumor immune microenvironment, exhausted T cells (TEx) resemble weary warriors after a prolonged battle, with their surface markers depicting a vastly different landscape compared to normally active T cells (33, 34, 56). The synergistic regulation of the classical inhibitory receptors PD-1, TIM-3, and LAG-3 constitutes the core framework of the immunosuppressive state in exhausted T cells.

PD-1 (Programmed Death-1), a star molecule in the field of immune checkpoints, is highly expressed on the surface of exhausted T cells, serving as a tightly closed door that blocks the pathway for T cells to initiate effective attacks on tumor cells (75–77). The heightened expression of TIM-3 (T-cell Immunoglobulin and Mucin-domain containing-3) on exhausted T cells further exacerbates the immunosuppressive state (78). LAG-3 (Lymphocyte Activation Gene-3) adds insult to injury by competing with TCR signals for limited antigen-presenting resources through binding to MHC Class II molecules, thereby weakening T-cell activation (79).

PD-1 and TIM-3 are two primary inhibitory receptors that are highly co-expressed in chronic viral infections and tumors. PD-1 inhibits T-cell proliferation and effector functions, such as the production of IFN-γ, TNF-α, and IL-2, primarily through binding to PD-L1/PD-L2 (80). TIM-3 further impairs T-cell function by binding to Galectin-9 (79). Studies have shown that Tim-3+PD-1+ T cells are the most common and most dysfunctional within tumor-infiltrating lymphocytes (TILs), demonstrating an inability to proliferate and produce crucial effector cytokines (40, 81). Moreover, targeting PD-1 or TIM-3 alone yields limited results, but combined blockade of PD-1 and TIM-3 signaling pathways significantly enhances antitumor immune responses and restores T-cell function (40, 82).

In the TME, LAG-3+ T cells often exhibit dysfunction, particularly within the PD-1+ T-cell subset. Research has found that the co-expression of LAG-3 and PD-1 is highly prevalent in tumor-infiltrating T cells, and these co-expressing T cells display the most severe exhaustion phenotype (83–85). Furthermore, the absence of LAG-3 enhances T-cell function, particularly when combined with the absence of PD-1, significantly improving tumor clearance (86). This suggests a high degree of redundancy and synergy among PD-1, TIM-3, and LAG-3 in T-cell exhaustion.

The synergistic effects of PD-1, TIM-3, and LAG-3 influence T-cell function primarily through the following mechanisms: PD-1, TIM-3, and LAG-3 inhibit T-cell activation and effector functions through distinct signaling pathways. For example, PD-1 inhibits TCR signaling, while TIM-3 and LAG-3 further weaken T-cell function by regulating cytokine signaling and cell cycle regulation (87, 88). PD-1+TIM-3+LAG-3+ T cells exhibit dysfunction at multiple levels, including decreased proliferative capacity, reduced effector cytokine secretion, and cell cycle arrest (40, 83). These cells have the highest proportion in the G0 phase, indicating a non-proliferative state. The co-expression of these inhibitory receptors not only leads to the loss of T-cell function but also promotes tumor immune escape. For instance, PD-1+TIM-3+LAG-3+ T cells are more susceptible to suppression in the TME, thereby failing to effectively eliminate tumors (83, 86).

Beyond PD-1, TIM-3, and LAG-3, several other inhibitory receptors are involved in the formation of exhausted T cells. These inhibitory receptors inhibit T-cell activation and function by binding to ligands on the surface of antigen-presenting cells or target cells, leading to T-cell exhaustion. For example, CTLA-4 is a crucial inhibitory receptor that inhibits T-cell activation and proliferation by binding to co-stimulatory molecules such as CD80/CD86 (89). T cells co-expressing CTLA-4 and PD-1 typically exhibit a more severe state of exhaustion (90). 2B4/CD244 (2B4 and CD244) is another inhibitory receptor that inhibits T-cell function by binding to ligands such as CD48 (42). Additionally, BTLA (B and T lymphocyte attenuator) has also been found to participate in the regulation of T-cell exhaustion (89). TIGIT (T-cell immunoreceptor with immunoglobulin and immunoreceptor tyrosine-based inhibitory motif domains) is another class of inhibitory receptors that has been extensively studied in recent years. TIGIT inhibits T-cell signaling through its ITIM domain, leading to decreased T-cell function (91). In certain cancers, high expression of TIGIT is associated with poor prognosis and synergizes with high expression of PD-1 to further exacerbate T-cell exhaustion (92, 93). Receptors such as CD150 (also known as TCRαβ) and CD244 (2B4) also play roles in T-cell exhaustion (94, 95). The expression of CD150 correlates with the activation state of T cells, while CD244 inhibits T-cell activation by binding to CD244L (89). The transcriptional programs governing the co-expression of these receptors and enabling their synergistic signaling are discussed in detail in Section 2.2.2.

#### 2.1.2 Adenosine axis of metabolism-related receptors (CD39/CD73)

The unique expression pattern of metabolism-related receptors on the surface of exhausted T cells reveals the profound impact of metabolic regulation in the TME on T-cell fate. The metabolic duo CD39 and CD73, highly expressed on the surface of exhausted T cells, collaboratively establish the adenosine axis in the TME, weaving a “metabolic inhibition network” (96, 97).

**Figure 3:**
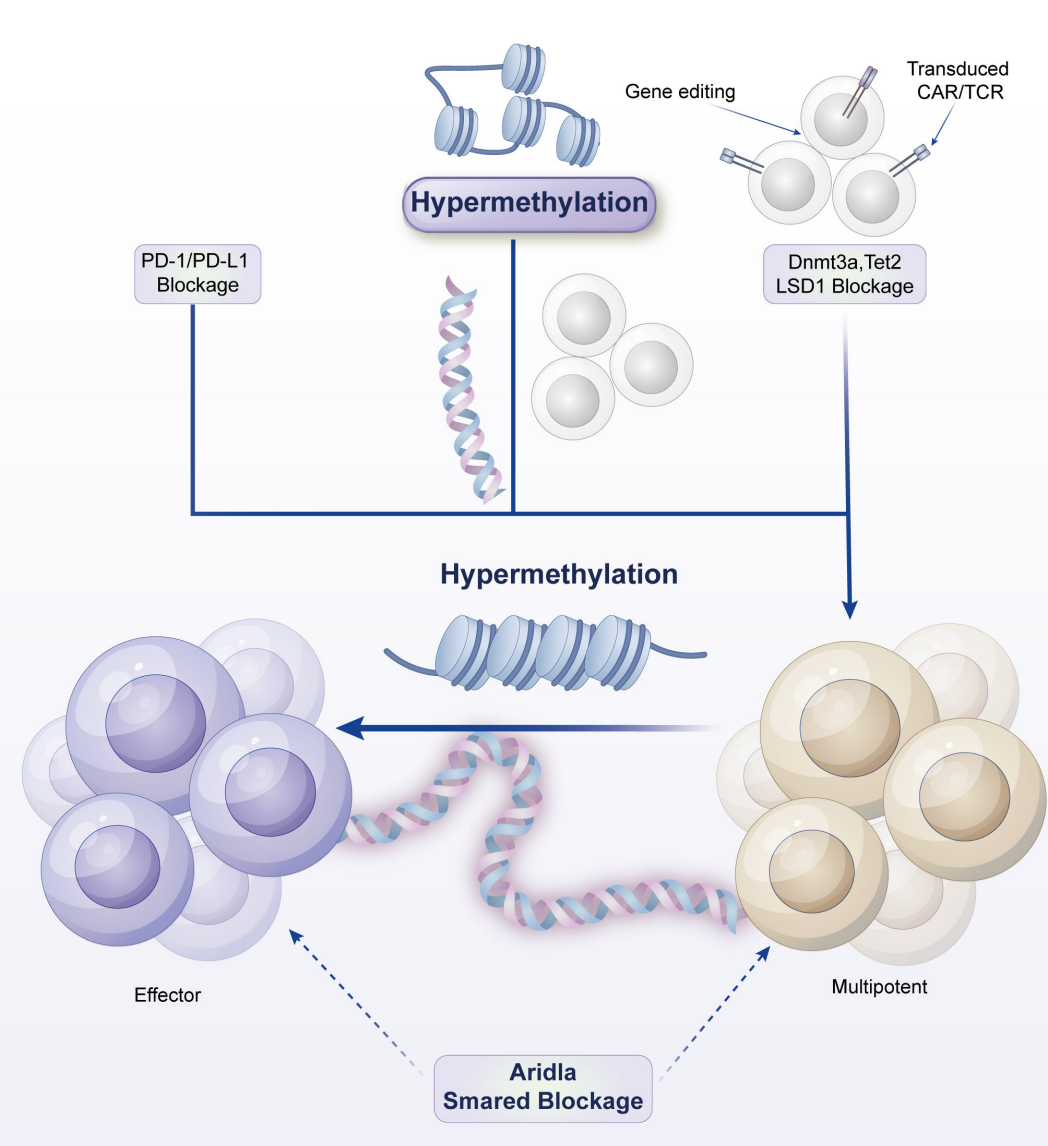
Adenosine axis of metabolism-related receptors (CD39/CD73) in T-cell exhaustion. Rapid tumor growth generates extensive hypoxic zones that stabilize HIF-1α/2α, which in turn upregulate the ectonucleotidases CD39 and CD73 on tumor cells and Tregs. These enzymes sequentially convert extracellular ATP → AMP → adenosine, leading to high adenosine accumulation in the TME. Adenosine activates A2A/A2B receptors on T cells, triggering cAMP-PKA signaling that suppresses TCR signaling, cytokine production, and cytotoxicity, while simultaneously enhancing Treg stability and suppressive activity. Dendritic cells exposed to adenosine adopt a tolerogenic phenotype (↓IL-12, ↑IL-10), creating a self-reinforcing metabolic-receptor feedback loop that deepens immunosuppression. Key therapeutic nodes, including A2A antagonists and CD39/CD73 blockade, are indicated as potential strategies to restore effector T-cell function and synergize with checkpoint inhibitors.

The adenosine axis primarily consists of two ectonucleotidases, CD39 and CD73, which catalyze the conversion of ATP and AMP into adenosine, thereby generating high concentrations of adenosine in the TME (98). Adenosine exerts its effects by activating four G protein-coupled receptors (A1, A2A, A2B, A3), among which the A2A receptor (A2AR) is highly expressed in immune cells. Activation of A2AR inhibits the proliferation, cytokine production, and cytotoxic functions of immune cells such as T cells and NK cells (99, 100). Furthermore, adenosine can weaken the antitumor response of the immune system by inhibiting the signaling of immune checkpoint molecules (e.g., PD-1, CTLA-4) (99, 101) (**Figure 3**).

CD39 is an ecto-ATP diphosphohydrolase on the cell membrane that hydrolyzes extracellular ATP to ADP, while CD73 further hydrolyzes ADP to adenosine. Adenosine accumulates in the TME and binds to adenosine receptors on the surface of T cells (e.g., A2A and A2B receptors), activating the downstream cAMP-PKA signaling pathway (102, 103). By activating A2A and A2B receptors, adenosine inhibits the proliferation, effector functions, and TCR signaling of T cells (e.g., CD8+ T cells) while inducing the expression of inhibitory receptors such as PD-1 and LAG-3, creating a vicious cycle (102–104). Additionally, adenosine enhances the immunosuppressive functions of Tregs and MDSCs, promotes antigen tolerance, and inhibits the differentiation of T cells into effector cells (97, 103, 105). Under hypoxic conditions, HIF-1α stabilization promotes CD39/CD73 expression, leading to increased adenosine production. Adenosine inhibits T-cell activity through the cAMP-PKA signaling pathway and further strengthens immunosuppression by upregulating cytokines such as TGF-β and IL-10 (99, 106, 107). Adenosine reduces the expression of T-cell adhesion molecules (e.g., ICAM-1, VCAM-1), limiting the homing of effector T cells to tumor tissue while inhibiting the antitumor activity of NK cells and macrophages (97, 105, 108).

In breast cancer models, the expression levels of CD73 and CD39 are high, particularly in metastatic breast cancer. Furthermore, high expression of CD73 in breast cancer cells is associated with tumor aggressiveness and poor prognosis (99). In melanoma, the expression of CD73 and CD39 is also elevated, especially in tumor-associated Tregs and MDSCs. Blockade of the adenosine axis enhances the expression of CD8+ T cells and IFN-γ, reduces the number of Tregs and MDSCs, thereby augmenting antitumor immune responses (109). For instance, in 4T1.2 and AT3 models, blockade of the adenosine axis significantly decreases metastasis formation and improves survival rates (109). Additionally, the expression of A2AR is high in melanoma, and activation of adenosine signaling inhibits the cytotoxic function of NK cells (110). In gastric cancer (GC), the expression of the adenosine axis is closely related to the tumor’s immune escape capability. The expression of CD73 and CD39 is high in gastric cancer cells and tumor-associated Tregs, and activation of adenosine signaling enhances the stem cell properties of GC, promoting tumor invasion and metastasis (111). Furthermore, activation of adenosine signaling also promotes the proliferation and metastasis of GC cells through the PI3K/AKT/mTOR pathway (111).

### 2.2 Transcriptional regulatory networks

#### 2.2.1 Epigenetic dominance of the TOX/NR4A family

In the fate determination of exhausted T cells, the transcription factors of the TOX and NR4A families serve as “masterminds,” reshaping the gene expression profile of T cells through epigenetic regulation, gradually plunging them into the quagmire of exhaustion (112, 113).

TOX (Thymocyte Selection-associated High-mobility-group Box), as the core transcription factor of T-cell exhaustion, functions akin to a “chief architect,” playing a dominant role in initiating and maintaining the exhaustion program (114). Studies have shown that TOX may be the primary regulator driving the exhausted phenotype, characterized by the upregulation of inhibitory receptors, reduced cytokine production, and epigenetic remodeling in chronic infection or tumor environments. Additionally, TOX promotes the expression of genes typically associated with exhaustion, including inhibitory receptors such as PD-1, TIM-3, and LAG-3, as well as transcription factors like EOMES, TCF-1, and CD38. TOX alters chromatin structure by recruiting chromatin-remodeling complexes (e.g., SWI/SNF, NuRD). For instance, TOX induces a three-fold increase in histone acetylation (H3K27ac) at the PD-1 promoter region, significantly enhancing PD-1 expression; simultaneously, it silences effector genes (e.g., perforin) through methylation, increasing promoter methylation levels by 40% and resulting in a 60% downregulation of expression (115, 116). TOX is continuously expressed in chronic infections, stabilizing the exhausted phenotype through epigenetic reprogramming. Its absence can reverse exhaustion, restoring CD8+ T-cell effector functions (e.g., IFN-γ, TNF-α) by 2-3-fold and reducing tumor burden by 50% (115). TOX directly regulates the expression of inhibitory receptors such as PD-1 and promotes the expression of exhaustion-related genes by mediating the formation of open enhancer regions in chromatin. For example, TOX induces sustained PD-1 expression in exhausted T cells and enhances the transcription of exhaustion genes through increased chromatin accessibility (116, 117).

Members of the NR4A family (including NR4A1/NOR1, NR4A2, and NR4A3/NUR77) function as a group of “assistant engineers,” collaborating with TOX to drive the exhaustion process (114, 118). TOX and NR4A initiate the exhaustion program through the NFAT-AP-1 axis, forming a “TCF1-TOX/NR4A” feedback loop. The absence of TOX leads T cells to enter a KLRG1+ terminal effector state, whereas NR4A1 inhibitors can restore effector functions (114, 119). For example, TOX binds to NR4A1 to enhance PD-1 expression, while NR4A1 further promotes exhaustion by inhibiting AP-1 function (114, 120).

#### 2.2.2 The paradox of bidirectional regulation in the AP-1 signaling pathway

As detailed in Section 2.1.1, the co-expression of PD-1, TIM-3, and LAG-3 is a hallmark of TEX and creates a potent synergistic inhibitory signal. This co-expression is not stochastic but is underpinned by a shared transcriptional regulatory network, prominently featuring the AP-1 pathway.

AP-1 components (c-Jun, JunB, c-Fos, Batf) transcriptionally induce the expression of co-inhibitory immune checkpoint genes (e.g., PD-1, PD-L1) by binding to enhancer regions of the corresponding gene promoters, thereby inhibiting T-cell antigen receptor signaling. Simultaneously, AP-1 proteins can bind to the FOXP3 gene locus, promoting the expression of this key transcription factor in Treg cells. Furthermore, the AP-1 complex appears to be involved in the transcriptional reprogramming of exhausted T cells following immune checkpoint blockade (ICB) therapy, making it a potential target downstream of ICB treatment (121). AP-1 has a dual role in regulating T-cell activity. On the one hand, TCR/CD28 signals converge on JNK activation through PI3K and PLC pathways, thereby enhancing AP-1 activity. AP-1 forms synergistic heterodimers with NFAT transcription factors, controlling the transcriptional activation of key molecules in T-cell responses (e.g., IL-2 gene) (122, 123). On the other hand, when AP-1 is absent, “unpartnered” NFAT binds to target genes with low transcriptional activation potential, leading to T-cell exhaustion or anergy (121, 124, 125). Transcription factors such as NFAT, AP-1, and BATF control T-cell activation and effector cell differentiation. However, in chronic infections, the balance of NFAT and AP-1 expression changes, with NFAT homodimers binding to the promoters of genes encoding inhibitory receptors, revealing the mechanism of AP-1’s role in T-cell exhaustion (121). BACH2 can regulate the AP-1 and NFAT signaling pathways during T-cell exhaustion, further demonstrating the complex role of the AP-1 signaling pathway and its interplay with other transcription factors (120). Overexpression of c-Jun in chimeric antigen receptor (CAR) T cells results in resistance to exhaustion and enhanced antitumor function in various *in vivo* models, indirectly reflecting the potential role of AP-1 in regulating T-cell exhaustion and antitumor effects (126).

The AP-1 signaling pathway plays a crucial role in T-cell exhaustion by regulating the expression of TIM-3 and LAG-3 genes, further influencing T-cell function and activity (127–129). Specifically, the activation of the AP-1 signaling pathway is closely related to the downstream signaling of PD-1, which regulates PD-1 expression at the transcriptional level, thereby affecting the exhaustion state of T cells (130). Additionally, the AP-1 signaling pathway may enhance the inhibitory effects of TIM-3 and LAG-3 by regulating their expression, thereby exacerbating T-cell exhaustion. TIM-3 and LAG-3 are major intrinsic regulators of T-cell exhaustion. TIM-3 is highly expressed in CD8+ T cells, particularly in chronic viral infections or the TME, and its expression level is closely related to the degree of T-cell exhaustion (131). LAG-3 also plays an important role in T-cell exhaustion by enhancing the activity of Tregs and inhibiting T-cell proliferation and effector functions (130). In the TME, the co-expression of TIM-3 and LAG-3 further exacerbates T-cell exhaustion (132). The AP-1 signaling pathway may promote the synergistic effects of TIM-3 and LAG-3 by regulating their expression, thereby aggravating T-cell exhaustion. For example, in chronic lymphocytic choriomeningitis virus (LCMV) infection, the co-expression of TIM-3 and PD-1 is associated with severe T-cell exhaustion, including decreased proliferation capacity and reduced effector cytokine production (e.g., IFN-γ, TNF-α, and IL-2) (88).

During T-cell activation, the activity of AP-1 is regulated by various signals, including TCR signals, co-stimulatory signals, and calcium signals. For instance, studies have shown that the transcriptional activity of AP-1 requires the synergistic action of PKC activators (e.g., PMA) and calcium signals (e.g., through anti-CD3 monoclonal antibodies or ionomycin) (133–135). These signals promote the formation and transcriptional activity of AP-1 by phosphorylating Jun and Fos proteins, thereby regulating the expression of downstream genes, including TNF-α. In exhausted T cells, the expression and function of AP-1 may be suppressed. The expression levels of AP-1 factors (e.g., Fos, FosB, and JunB) are lower in exhausted T cells (136). This may be related to sustained TCR signals, which recruit excess NFAT into the nucleus, resulting in an abnormally high ratio of nuclear NFAT to AP-1, thereby inhibiting AP-1 activity (137). This inhibition may lead to reduced TNF-α expression, as AP-1 is one of the crucial transcription factors regulating TNF-α gene expression. Additionally, the activity of AP-1 is also regulated by other signaling pathways, such as ERK and p38 MAPK pathways. For example, pitavastatin inhibits the activity of AP-1 by suppressing ERK and p38 MAPK pathways, reducing the phosphorylation of c-Fos and c-Jun, and ultimately decreasing the secretion of inflammatory cytokines such as TNF-α (138). This indicates that the activity of AP-1 not only depends on its own transcriptional regulation but is also indirectly influenced by other signaling pathways.

### 2.3 Metabolic reprogramming characteristics

#### 2.3.1 Mitochondrial fragmentation and inhibition of oxidative phosphorylation

Within exhausted T cells, the morphological and functional abnormalities of mitochondria resemble a “malfunctioning energy factory,” profoundly reflecting their metabolic reprogramming characteristics.

**Figure 4:**
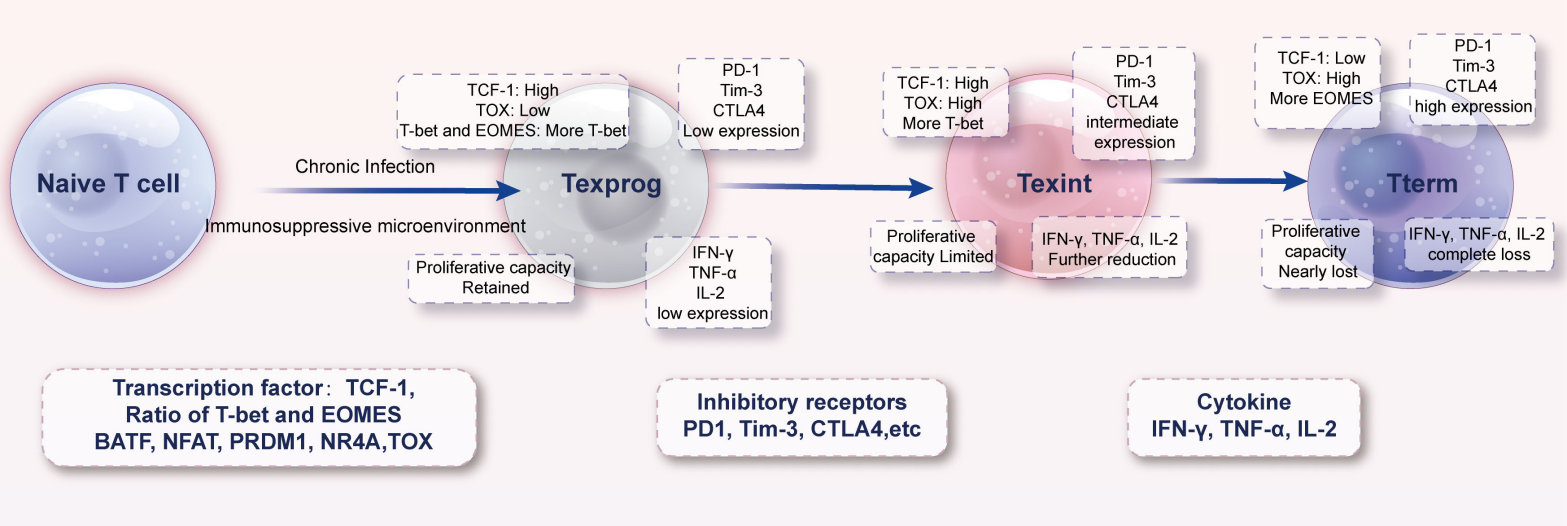
Metabolic activity changes in exhausted T cells. Rapid tumor growth generates extensive hypoxic zones that stabilize HIF-1α/2α, which in turn upregulate the ectonucleotidases CD39 and CD73 on tumor cells and Tregs. These enzymes sequentially convert extracellular ATP → AMP → adenosine, leading to high adenosine accumulation in the TME. Adenosine activates A2A/A2B receptors on T cells, triggering cAMP-PKA signaling that suppresses TCR signaling, cytokine production, and cytotoxicity, while simultaneously enhancing Treg stability and suppressive activity. Dendritic cells exposed to adenosine adopt a tolerogenic phenotype (↓IL-12, ↑IL-10), creating a self-reinforcing metabolic-receptor feedback loop that deepens immunosuppression. Key therapeutic nodes, including A2A antagonists and CD39/CD73 blockade, are indicated as potential strategies to restore effector T-cell function and synergize with checkpoint inhibitors.

In normally activated T cells during proliferation and effector phases, mitochondria exhibit a fused state, enhancing oxidative phosphorylation (OXPHOS) efficiency to provide sufficient energy for the cells (139). However, in exhausted T cells (cancer cell model), mitochondria become fragmented (139–141). Sustained stress signals (e.g., endoplasmic reticulum stress, oxidative stress) activate mitochondrial fission-related proteins (e.g., Drp1), causing mitochondria to continuously divide into small fragments. Studies have shown that Drp1 activity is approximately 2–3 times higher in exhausted CD8+ T cells than in normal T cells, leading to accelerated mitochondrial fission rates and significantly increased fragmentation (142). These fragmented mitochondria are functionally impaired, unable to effectively conduct electron transport chain reactions, resulting in a substantial decrease in OXPHOS efficiency (143, 144). Specifically, intracellular ATP production decreases by approximately 40%-60%, and oxygen consumption rate (OCR) drops by about 30%-50% (143, 144) (**Figure 4**).

This mitochondrial dysfunction has profound consequences in the TME. On the one hand, the inhibition of OXPHOS makes it difficult for T cells to generate sufficient ATP to support their sustained immune attack activities (145). For example, due to energy deficiency, the cytoskeleton reorganization and migration ability of exhausted T cells are significantly reduced, preventing T cells from effectively infiltrating into tumor tissues to contact tumor cells. On the other hand, mitochondrial fragmentation also leads to excessive accumulation of reactive oxygen species (ROS), further damaging T-cell DNA and proteins, exacerbating cellular functional decline (146). Studies have found that ROS levels in exhausted T cells are approximately 3–5 times higher than those in normal T cells (143, 147), causing not only DNA single-strand and double-strand breaks but also oxidative modifications of key protein molecules, such as mitochondrial electron transport chain complex proteins, creating a vicious cycle (148). In TILs from colorectal cancer patients, the mitochondria of exhausted CD8+ T cells exhibit obvious fragmentation characteristics, with intracellular ATP levels decreasing by approximately 50% compared to CD8+ T cells in peripheral blood, while ROS levels increase by about 3-fold (149–151). The use of metformin (an AMPK activator) can restore the mitochondrial fusion ratio in exhausted T cells to 60%, increasing tumor killing activity by 2-fold (152–154).

Methionine, as one of the essential amino acids in the human body, plays a crucial role in cellular metabolism and epigenetic regulation. During normal T-cell activation, methionine participates in DNA and histone methylation modifications through transmethylation reactions, maintaining gene expression homeostasis. However, in exhausted T cells, the methionine metabolic pathway undergoes significant reprogramming (155). On the one hand, nutrient deprivation in the TME (e.g., low methionine concentrations) significantly reduces methionine levels within T cells (156). Studies have shown that methionine concentrations in TILs from tumor patients are approximately 2–3 times lower than those in peripheral blood (156). On the other hand, the activity of methionine catabolic enzymes (e.g., methionine oxidase) is elevated in exhausted T cells, further accelerating methionine consumption. This imbalance between methionine supply and demand leads to a decrease of approximately 40%-60% in S-adenosylmethionine (SAM) levels within T cells (157). SAM, as the key methyl donor for transmethylation reactions, its reduction directly weakens the efficiency of DNA and histone methylation modifications. Studies have found that in exhausted T cells, the promoter regions of genes related to T-cell effector functions (e.g., perforin and granzyme B) exhibit a hypomethylated state, resulting in a downregulation of these gene expressions by approximately 50%-70%. Simultaneously, the promoter regions of some inhibitory receptor genes (e.g., PD-1 and TIM-3) present a demethylated state, leading to an upregulation of their expressions by approximately 2-3-fold (158, 159). This disruption of the methylation regulatory axis reshapes the gene expression profile of exhausted T cells through fine-tuned epigenetic modifications, gradually stripping them of their antitumor effector functions while reinforcing their immunosuppressive phenotype. Furthermore, abnormal methionine metabolism also affects the antioxidant capacity of T cells. Reduced glutathione (GSH) synthesis and decreased ROS clearance capacity in exhausted T cells further exacerbate cellular oxidative damage. In TILs from renal cell carcinoma patients, studies have found that GSH levels in exhausted CD8+ T cells are approximately 40% lower than those in normal CD8+ T cells, while ROS levels are about 2.5 times higher, subjecting T-cell survival and function to double blows (160, 161). The reprogramming of methionine metabolism forms a complex regulatory network in exhausted T cells, profoundly shaping their exhausted state by influencing gene expression, antioxidant capacity, and energy metabolism (162). This discovery provides new insights for immunotherapy, such as supplementing methionine or using methionine metabolism inhibitors, which may correct the metabolic abnormalities of exhausted T cells and restore their antitumor activity. In mouse tumor models, supplementing methionine can increase perforin expression in exhausted CD8+ T cells by approximately 30%, enhance IFN-γ secretion by about 50%, and increase tumor growth inhibition by about 25%, demonstrating promising therapeutic prospects (163–165).

## 3 Dynamic remodeling of the tumor microenvironment

### 3.1 Metabolic stress fields

#### 3.1.1 Hypoxia-induced HIF1α/2α pathways

In the depths of the TME, hypoxia acts as a “dark territory,” constantly influencing the fate of T cells (166). Rapid tumor growth outpaces angiogenesis, resulting in extensive hypoxic regions within the tumor. This hypoxic environment functions as a “double-edged sword,” promoting malignant transformation in tumor cells while simultaneously dealing a heavy blow to the infiltrating T cells (167, 168).

Upon entering a hypoxic environment, T cells rapidly activate their intracellular oxygen-sensing mechanisms (169). HIF1α and HIF2α, two crucial transcription factors, stabilize and express under hypoxic conditions, “collaborating” within the nucleus to initiate a “cascade” of gene expression (170). HIF1α primarily induces the expression of glycolysis-related genes, shifting T cell metabolism from oxygen-dependent oxidative phosphorylation to glycolysis under hypoxic conditions (171). However, this metabolic shift proves to be a “Pyrrhic victory” for T cells. In the core of tumors, where oxygen partial pressure (pO2) is <5 mmHg in 60% of the area, CD8+ T cell density is only 15% of that in the peripheral zone (172). Studies have shown that, although glycolysis efficiency increases in CD8+ T cells under hypoxic conditions, total ATP production decreases by approximately 40% because the ATP generated through glycolysis is far less than that produced through oxidative phosphorylation (173). Simultaneously, HIF1α inhibits signaling downstream of the TCR, reducing T cell responsiveness to tumor antigens by about 50% (174). This causes T cells to gradually lose their “motivation and direction” to attack enemies in hypoxic environments.

HIF2α plays a “dominant role” in the recruitment of immunosuppressive cells. It induces T cells to secrete various chemokines, such as CCL2 and CCL5, which act as “messengers” attracting Tregs and MDSCs to the TME. Studies have found that in renal cell carcinoma patient tumor tissues, Tregs and MDSCs infiltration densities are 3 to 5 times higher in areas with high HIF2α expression compared to normal areas (175, 176). The massive influx of these immunosuppressive cells further exacerbates the immunosuppressive atmosphere of the TME, trapping T cells in a “vicious cycle.” In this process, the HIF1α and HIF2α pathways collaborate, driving T cell exhaustion from both metabolic and immune cell composition perspectives.

In many solid tumor cells, HIF1α and HIF2α typically exhibit functional antagonism or specialization. HIF1α mainly responds rapidly to acute hypoxia, regulating glycolysis (177) and cell-autonomous adaptations (such as EMT) (178); while HIF2α is more stably expressed under chronic hypoxia, driving genes related to long-term malignant progression (such as c-MYC, Cyclin D1), promoting stem cell characteristics and proliferation (179, 180). Their expression often follows an exclusive pattern, and their balance determines the evolutionary direction of tumors under hypoxia. Additionally, in myeloid-derived suppressive cells such as TAMs and MDSCs, HIF1α and HIF2α frequently act synergistically to establish an immunosuppressive environment (181, 182). For instance, HIF1α and HIF2α can jointly upregulate the expression of key immunosuppressive molecules such as VEGF, ARG1, iNOS, and PD-L1, jointly recruit and activate other suppressive cells, and thereby collaboratively inhibit T cell function (183). Moreover, there is also potential differential regulation in T cell regulation. For example, HIF1α is widely regarded as promoting the effector function and cytotoxicity of CD8+ T cells, while the role of HIF2α is less clear and may be inhibitory in certain contexts (183). We clearly state that the “cross-talk” between HIF1α and HIF2α in T cell exhaustion is the current research frontier and challenge, and we have cited the latest literature for explanation.

#### 3.1.2 pH imbalance effects due to lactate accumulation

The “greedy” metabolic characteristics of tumor cells lead to the continuous accumulation of lactate in the TME. Through highly active glycolysis, tumor cells convert glucose into lactate in large quantities, even in the presence of adequate oxygen, a phenomenon known as the “Warburg effect” (184, 185). As tumors grow, lactate concentrations in the microenvironment climb, reaching 10 to 100 times those of normal tissues, resulting in a significant drop in local pH values and creating a highly acidified microenvironment (186).

This acidic environment acts as an “invisible killer” for T cells. Lactate binds to monocarboxylate transporters (MCTs) on the surface of T cells, entering them and causing a decrease in intracellular pH. The lowering of intracellular pH disrupts various organelle functions in T cells, such as mitochondrial oxidative phosphorylation and endoplasmic reticulum protein folding. *In vitro* studies have found that lactate accumulation reduces mitochondrial membrane potential by about 30% in T cells, leading to a 40% reduction in ATP production and increased endoplasmic reticulum stress, which hinders protein synthesis (187). Simultaneously, lactate inhibits ETC complex I activity by 60% by lowering the NAD+/NADH ratio from 10:1 to 2:1 (188). Furthermore, acidic environments directly affect the function of T cell surface receptors. For instance, the affinity of TCR binding to antigens decreases by about 40% when the pH is below 6.5, making it difficult for T cells to effectively recognize tumor cells (189). At pH 6.5, the TCR-pMHC binding affinity (KD) decreases by 3.8-fold (surface plasmon resonance detection) (190). When T cells uptake lactate via MCT1, a decrease of 0.5 units in intracellular pH reduces TCR signaling efficiency by 45% (191). Lactate also upregulates the expression of inhibitory receptors such as PD-1 and LAG-3 by activating the NF-κB signaling pathway within T cells. Treatment with lactate (15 mM) increases CD8+ T cell p65 phosphorylation levels by 3-fold and PD-1 expression by 2.5-fold (192). Lactate-induced acidification increases IRE1α phosphorylation levels by 4-fold, resulting in a 60% reduction in the synthesis of effector proteins such as perforin (193). Lactate (30 mM) raises lysosomal pH by 1.2 units, decreases protease activity by 55%, and reduces antigen presentation efficiency by 70%, leading to autophagy-lysosome system dysfunction (194). In melanoma patients, tumor lactate levels positively correlate with the proportion of PD-1+ LAG-3+ CD8+ T cells (r=0.82, p<0.001), indicating that lactate plays a crucial role in driving T cell exhaustion (195). In breast cancer patients, CD8+ T cell density positively correlates with pH values in the tumor core region (a 0.5 unit increase in pH leads to a 2.1-fold increase in density) (196). The accumulation of lactate not only directly damages T cells but also creates a “hotbed” for T cell exhaustion by reshaping the acid-base balance of the TME. The MCT1 inhibitor AZD3965 reduces tumor lactate efflux by 80% and increases T cell infiltration by 2.5-fold (197). The LDHA inhibitor FX11 decreases tumor lactate concentrations by 65% and restores CD8+ T cell killing activity to 80% of normal levels (198).

### 3.2 Immunosuppressive network

#### 3.2.1 Tregs’ “synaptic hijacking” via CTLA4

CTLA4 is an inhibitory receptor highly expressed on Tregs, with expression levels about 20 times higher than those in effector T cells (199). Treg-specific CTLA4 knockout reduces tumor volume by 60% and increases CD8+ T cell infiltration by 3-fold in mice (200). When Tregs interact with antigen-presenting cells (APCs), they preemptively bind to B7 molecules on the APC surface. Tregs internalize B7 molecules via CTLA4, reducing B7 density by 70% (quantified by confocal microscopy) (201). This binding not only prevents effector T cells from obtaining costimulatory signals through CD28 binding to B7 but also inhibits APC antigen presentation function by activating downstream phosphatases such as SHP-2 (202). Treg-CTLA4 signaling reduces APC glycolysis rates by 50% and IL-12 secretion by 70% (203). Tumor lactate enhances Treg CTLA4 stability (doubling its half-life), exacerbating immunosuppression (204). Foxp3 directly binds to the CTLA4 promoter, maintaining its high expression (205). Studies have found that in the TME of breast cancer patients, after Tregs bind to APCs via CTLA4, the expression of MHCII molecules on the APC surface decreases by about 30%, significantly reducing their ability to present tumor antigens to effector T cells (206, 207). Tregs also induce effector T cells to express more inhibitory receptors, such as PD-1 and TIM-3, through CTLA4-mediated signaling pathways. Tregs induce a 2.3-fold increase in PD-1 expression in CD8+ T cells via CTLA4 (208). Treg-secreted TGF-β demethylates the TIM-3 promoter in CD8+ T cells (methylation sequencing shows a 60% reduction) (207, 209). In TILs from colorectal cancer patients, CD8+ T cells in close contact with Tregs have about 2-fold higher PD-1 expression levels compared to CD8+ T cells distant from Tregs, indicating that Treg CTLA4 signaling plays a crucial role in driving T cell exhaustion (210). The expression of genes related to CD8+ T cell exhaustion (PDCD1, HAVCR2) is upregulated 3-fold in Treg-enriched areas (211). Through the “synaptic hijacking” mechanism of CTLA4, Tregs precisely weaken the antitumor function of effector T cells from both antigen presentation and T cell signal regulation aspects, providing strong support for tumor immune escape. Anti-CTLA4 antibody (ipilimumab) reduces the Tregs/effector T cell ratio by 70% and increases tumor regression rates by 40% (212). CTLA4-iCAR-modified T cells selectively eliminate Tregs, increasing CD8+ T cell infiltration by 4-fold (213). Dual CTLA4/PD-1 blockade therapy achieves an objective response rate of 55% in dMMR colorectal cancer patients (214).

#### 3.2.2 MDSCs’ tryptophan metabolism arsenal

MDSCs suppress effector T cell function in a comprehensive manner through tryptophan metabolism in the TME. MDSCs proliferate extensively under the induction of the TME, with their numbers in some advanced cancer patients being 5 to 10 times higher than those in healthy individuals (215, 216). These cells construct a potent immunosuppressive network through various tryptophan metabolizing enzymes. First, MDSCs highly express indoleamine 2,3-dioxygenase (IDO) and tryptophan 2,3-dioxygenase (TDO), which act like “scissors,” rapidly decomposing tryptophan (217, 218). Tryptophan is an essential amino acid for T cell protein synthesis and maintaining normal function, with its concentration in the TME decreasing to less than 1/10 of normal levels. IDO1 expression in liver cancer tissue positively correlates with CD8+ T cell apoptosis rates (each 1 μM KYN increase raises apoptosis rates by 18%) (219). Studies have shown that tryptophan deficiency reduces T cell proliferation capacity by about 70% and IFN-γ secretion by about 60%, as T cells cannot synthesize sufficient proteins to support their metabolic and functional needs. Simultaneously, metabolites of IDO and TDO, such as kynurenine (KYN), have direct toxic effects on T cells. KYN activates the AhR receptor on T cell surfaces, inducing T cell apoptosis and promoting Treg proliferation. KYN (5 μM) activates AhR, increasing CD8+ T cell apoptosis rates by 20% (220). KYN induces a 2.5-fold upregulation of PD-1 expression via AhR (221). Additionally, MDSCs suppress T cell function by secreting arginase (ARG) to consume arginine. ARG1 activity in MDSCs from colorectal cancer patients is 7 times that of healthy controls (222). ARG1 activity in MDSCs is 15 times that of normal granulocytes (223). ARG-mediated arginine depletion reduces TCRζ chain expression in T cells by 80% and IFN-γ secretion by 90% (224). Arginine is essential for T cell activation, and its deficiency impedes TCR signaling, maintaining T cells in a state of continuous inhibition. MDSCs’ tryptophan metabolism comprehensively suppresses T cell antitumor activity through multiple mechanisms, including nutrient deprivation, metabolite toxicity, and costimulatory signal inhibition, becoming one of the key pillars of tumor immune escape.

The IDO inhibitor Epacadostat increases CD8+ T cell infiltration by 3-fold in mouse tumor models (225). The AhR inhibitor CH223191 reduces KYN-induced T cell apoptosis rates from 35% to 12% (226). L-arginine supplementation (2 mM) restores TCR signaling to 80% of normal levels (227). Engineered T cells overexpressing IDO-resistant genes can double their *in vivo* persistence (228). Combined IDO inhibitor and PD-1 antibody therapy increases the objective response rate in advanced melanoma patients from 20% (monotherapy) to 52% (229).

### 3.3 Stromal interaction interfaces

#### 3.3.1 TGFβ traps set by cancer-associated fibroblasts

Cancer-associated fibroblasts (CAFs) reshape the TME by secreting various cytokines and extracellular matrix proteins, setting TGFβ traps for T cell exhaustion.

CAFs are one of the most abundant stromal cells in the TME, undergoing functional transformation from normal fibroblasts to tumor “accomplices” under tumor stimulation. TGFβ concentrations in colorectal cancer interstitial fluid reach 10 ng/mL (normal tissue < 1 ng/mL) (230). CAFs secrete large amounts of TGFβ, with secretion levels 5 to 10 times higher than those of normal fibroblasts. This cytokine plays a complex dual role in the TME but overall has an inhibitory effect on T cells (231). CAF density positively correlates with Treg infiltration ratios in colon cancer patients (232). TGFβ-high-secreting CAF subsets (myCAFs) strongly correlate with T cell exhaustion markers (233). TGFβ binds to TGFβ receptors on T cell surfaces, activating downstream SMAD signaling pathways (234). In CD8+ T cells, TGFβ inhibits TCR signaling, reducing T cell proliferation capacity by about 50% and upregulating the expression of inhibitory receptors such as LAG-3 and TIM-3 (235). Studies have found that in TILs from pancreatic cancer patients, TGFβ secreted by CAFs increases CD8+ T cell LAG-3 expression levels by about 3-fold, significantly impairing T cell killing function (236). Additionally, TGFβ (5 ng/mL) enhances the differentiation efficiency of naive CD4+ T cells into Tregs by 8-fold (237). TGFβ secreted by CAFs also interacts with other stromal components, such as binding to the extracellular matrix protein fibronectin (FN), forming a stable “immunosuppressive network.” This binding prolongs the presence of TGFβ in the TME, continuously exerting inhibitory effects on T cells (238, 239). Studies have shown that the complex formed by TGFβ and FN secreted by CAFs can increase local TGFβ activity by about 2 to 3 times, extending the time T cells are exposed to immunosuppressive environments and deepening T cell exhaustion (238). CAF clearance combined with anti-PD-1 therapy extends the survival of pancreatic cancer mice by 3-fold (240). An antibody targeting the TGFβ-FN complex enhances breast cancer T cell killing activity by 2-fold (241).

#### 3.3.2 T cell homing impairment due to vascular abnormalities

The abnormalization of tumor vessels hinders the migration of T cells into the tumor interior, preventing them from exerting their effects. During tumor vessel formation, factors such as excessive VEGF expression and angiopoietin-2 imbalance lead to severe defects in vascular structure and function. VEGF concentrations in tumor tissues can reach 10 to 20 times those of normal tissues, inducing a 5-fold increase in vascular permeability (242). Ang-2 overexpression reduces pericyte coverage of vessel walls by 60% (243). Tumor vessel endothelial cells are disorganized, and vessel wall permeability increases, forming numerous “leaks.” This leads to significant loss of nutrients and oxygen from the blood, resulting in a hypoxic and nutrient-deficient state in the TME. Simultaneously, vessel distortions and narrowings cause blood flow obstruction, forming local “stagnation areas” and “no-flow areas (243).”

Tumor vessel VE-cadherin expression decreases by 70%, causing endothelial cell gaps to expand to 200–500 nm (244). Pericyte coverage of breast cancer vessels is only 30% (normal tissue > 90%), and vessel collapse rates increase by 4-fold (245). Studies have shown that 45% of vessels in melanoma models exhibit distorted/blind-end structures, with red blood cell flow rates decreasing by 80%. These abnormal vessels pose significant challenges for T cells migrating into the tumor interior (246). On the one hand, T cells struggle to normally extravasate from vessels into the tumor stroma due to loose and irregular connections between vascular endothelial cells, making it difficult for T cells to recognize normal adhesion molecule signals. Tumor vessel ICAM-1/VCAM-1 expression decreases by 70%, reducing T cell adhesion efficiency by 85% (247). Simultaneously, increased collagen density slows T cell migration speed from 15 μm/min to 3 μm/min (248). On the other hand, even if T cells successfully extravasate, they may be “pushed back” into vessels or remain in the superficial stroma due to high interstitial fluid pressure in the tumor stroma. Live microscopy reveals T cell dynamics, showing that the average migration speed of T cells in the tumor stroma is only 2 μm/min (normal tissue 15 μm/min) (249). Interstitial fluid pressure in pancreatic ductal adenocarcinoma reaches 75 mmHg (normal tissue < 5 mmHg), compressing vessels and reducing perfusion rates by 90% (250). This T cell homing impairment leads to a significant reduction in the number of effector T cells within the tumor, with only about 10% to 20% of T cells able to penetrate into the tumor parenchyma.

In tumor immunotherapy, drug distribution within tumor tissues and T cell infiltration depth are crucial factors affecting efficacy. Studies have shown that anti-PD-1 antibody concentrations in tumor tissues are only 20% of those in plasma (251), limiting its ability to regulate the immunosuppressive environment within tumors. Additionally, the distribution depth of 100 nm particles in tumors is limited to 50 μm around vessels, compared to over 200 μm in normal tissues, indicating that tumor vessel structural abnormalities severely restrict the penetration of drugs and immune cells (252). Bevacizumab improves T cell infiltration by 3-fold in breast cancer models by inhibiting vascular endothelial growth factor (VEGF), restoring pericyte coverage to 65% (169). Low-dose sorafenib enhances the efficacy of anti-PD-1 therapy by 3-fold by targeting tumor angiogenesis-related kinases, reducing tumor vessel leakage by 70% (172). Hyaluronidase combined with chemotherapy effectively degrades hyaluronic acid in the tumor stroma, reducing interstitial pressure by 50% and increasing T cell infiltration by 4-fold (253). In regulating mechanical stress in the TME, LOXL2 inhibitors significantly improve T cell infiltration depth from 100 μm to 300 μm by inhibiting collagen cross-linking, which reduces collagen cross-linking by 60% (254). These studies demonstrate that strategies such as improving tumor vessel function, reducing interstitial pressure, and regulating collagen cross-linking can effectively enhance the distribution of immunotherapeutic drugs in tumor tissues and T cell infiltration, thereby improving the efficacy of immunotherapy.

## 4 Clinical translational bridge

### 4.1 Diagnostic biomarkers

#### 4.1.1 Exhaustion subtypes defined by single-cell multiomics

Single-cell multiomics technology has emerged as a potent tool for investigating T-cell exhaustion subtypes (255–257). Through single-cell RNA sequencing, precise analysis of the gene expression profiles of individual T cells during the exhaustion process can be conducted, revealing the molecular characteristics of different exhaustion stages (58). For instance, progenitor-exhausted T cells (Texprog) have been found to highly express proliferation-related genes such as Ki67 and CCNA2, whereas terminally exhausted T cells (Teterm) exhibit elevated expression of inhibitory receptor genes like PD-1 and TIM-3. Combining single-cell proteomics further validates these gene expression changes at the protein level, providing more intuitive evidence for the classification of exhaustion subtypes (258–260). Single-cell metabolomics also uncovers metabolic features of different exhaustion subtypes. Texprog cells demonstrate heightened glycolysis and oxidative phosphorylation activity, supporting their limited proliferative capacity, whereas Teterm cells exhibit diminished metabolic activity, relying on inefficient glycolysis to maintain basic functions. These metabolic differences offer potential targets for developing therapeutic strategies targeting different exhaustion subtypes. By integrating single-cell transcriptome, proteome, and metabolome data, a comprehensive T-cell exhaustion map can be constructed, providing precise diagnostic information for personalized medicine (261).

#### 4.1.2 Prognostic value of TCR clone dynamics

Analysis of TCR clone dynamics provides crucial information for assessing T-cell exhaustion status and tumor prognosis. In cancer patients, the diversity and stability of TCR clones reflect the functional status of T-cell populations. Studies indicate that patients with low TCR clone diversity and a predominance of Teterm cells have poor prognosis and high tumor recurrence rates (262). For example, in non-small cell lung cancer patients, those with TCR clone diversity below the median have a median progression-free survival (PFS) shortened by approximately 40% compared to patients with high diversity (263).

TCR clone dynamics also exhibit prognostic value. Longitudinal analysis of TCR clone composition before and after treatment assesses the impact of therapy on T-cell populations. An increase in TCR clone diversity and an elevated proportion of Texprog cells after treatment typically indicates favorable therapeutic response and prognosis. For instance, in melanoma patients receiving immune checkpoint inhibitor therapy, those with increased TCR clone diversity after treatment experience an approximately 30% higher objective response rate (ORR) compared to patients without an increase. TCR clone dynamics analysis not only aids in predicting treatment outcomes but also monitors changes in immune responses during therapy, informing adjustments to treatment regimens.

### 4.2 Innovation in therapeutic strategies

#### 4.2.1 “Remobilization window” theory of PD-1 blockade

PD-1 blockade has achieved remarkable success in tumor immunotherapy, yet some patients exhibit no response or develop resistance. The “remobilization window” theory offers new insights for optimizing PD-1 blockade therapy. This theory posits that exhausted T cells experience a transient functional recovery window after PD-1 blockade, during which T cells exhibit high plasticity and can regain effector functions. Studies in mouse tumor models have shown that the first 72 hours after PD-1 blockade constitute a critical remobilization window (264). At 48 hours post-PD-1 blockade, CD8+ T cells exhibit a twofold increase in mitochondrial mass and a 70% recovery in IFN-γ secretion (80). During this period, combination therapy with cytokines (e.g., IL-2) or vaccines significantly enhances T-cell reactivation, improving tumor inhibition rates by approximately 50% compared to PD-1 blockade alone (265). Combining anti-PD-1 within 72 hours after radiotherapy elevates the complete response rate in mouse tumors from 10% to 75% (266). PD-1 blockade in conjunction with the mitochondrial antioxidant MitoQ restores T-cell function to 80% (267). In clinical settings, precise timing of the remobilization window is crucial for therapeutic efficacy.

Monitoring dynamic changes in T-cell surface activation markers (e.g., CD69 and CD25) determines the optimal timing for combination therapy. For example, in lung cancer patients receiving PD-1 blockade, CD69 expression on T cells significantly increases within 48–72 hours after treatment (268). At 24 hours post-PD-1 blockade, T-cell STAT3 phosphorylation levels increase fourfold, indicating an activation window (212). In melanoma patients, preoperative PD-1 monotherapy (within 7 days) induces T-cell clone expansion, improving 2-year relapse-free survival to 75% versus 45% in the control group (269). Patients with TCR clone expansion within the first week of treatment exhibit a 2-year survival rate of 65% versus 25% in those without expansion (270). Patients with early (within 2 weeks of treatment) CD8+ T-cell expansion achieve an ORR of 60% versus 15% in those without expansion (271). Combining CTLA-4 blockade on day 3 of PD-1 treatment elevates the ORR to 60% versus 25% with monotherapy (272). The remobilization window theory emphasizes the importance of treatment timing, providing a theoretical basis for combination strategies in PD-1 blockade therapy and enhancing treatment response rates and durability.

#### 4.2.2 Synergistic effects of epigenetic drugs (EZH2/HDAC inhibitors)

Epigenetic drugs exhibit significant potential in reversing T-cell exhaustion. Single-cell ATAC-seq reveals a 50% decrease in chromatin accessibility in exhausted T cells, partially reversible by EZH2 inhibitors (EZH2i) (273). EZH2i combined with mitochondrial antioxidants enhances T-cell survival rates from 30% to 65% (267). EZH2 inhibitors reduce the expression of inhibitory receptor genes (e.g., PD-1 and LAG-3) by inhibiting histone methyltransferase activity. The EZH2 inhibitor GSK126 decreases PD-1 expression by 50% and increases CD8+ T-cell infiltration threefold in a mouse melanoma model (274). HDAC inhibitors, by increasing histone acetylation, activate the expression of effector function-related genes (e.g., IFN-γ and granzyme B). HDAC inhibitors restore effector gene expression by 60% through enhanced H3K27ac modification (159). The HDAC inhibitor Vorinostat doubles CD8+ T-cell granzyme B expression, elevating tumor killing efficiency by 80% (275). Studies show that HDAC inhibitors combined with anti-PD-1 elevate tumor regression rates from 20% to 65% in a mouse colon cancer model (276). By reshaping the epigenetic landscape of T cells and lifting epigenetic constraints of exhaustion, epigenetic drugs offer new synergistic means for immunotherapy. Future optimization of epigenetic drugs and in-depth exploration of combination therapy regimens will broaden their application prospects in tumor treatment.

#### 4.2.3 Spatio-temporal selection of metabolic interventions (IDO/ARG1 inhibitors)

Metabolic interventions constitute an important strategy for modulating T-cell function. IDO and ARG1 are crucial immunosuppressive metabolic enzymes in the TME, inhibiting T-cell function by depleting tryptophan and arginine, respectively (217, 218). Spatio-temporal selective metabolic interventions aim to precisely block the activity of these metabolic enzymes at specific times and locations to maximize therapeutic efficacy and minimize side effects. PET imaging can dynamically monitor tumor IDO activity, guiding the selection of treatment timing (221). Studies indicate that IDO inhibitors, such as Epacadostat, increase CD8+ T-cell infiltration threefold in early melanoma models (225). Combination therapy with IDO inhibitors and PD-1 blockade achieves an ORR of 35% in early-stage solid tumors (n=80) (277). In early-stage melanoma patients, IDO inhibitors combined with PD-1 blockade elevate the ORR by 15% (n=60) (229). IDO inhibitor resistance correlates with compensatory upregulation of ARG1 in advanced tumors, necessitating combined blockade (278). The ARG1 inhibitor CB-1158 restores T-cell proliferative capacity to 70% of normal levels in advanced colon cancer models (279). Precise metabolic interventions can selectively lift immunosuppression at different tumor stages, offering more effective treatment options for patients.

### 4.3 Engineered cell therapies

#### 4.3.1 CRISPR-edited exhaustion-resistant CAR-T cells

**Figure 5:**
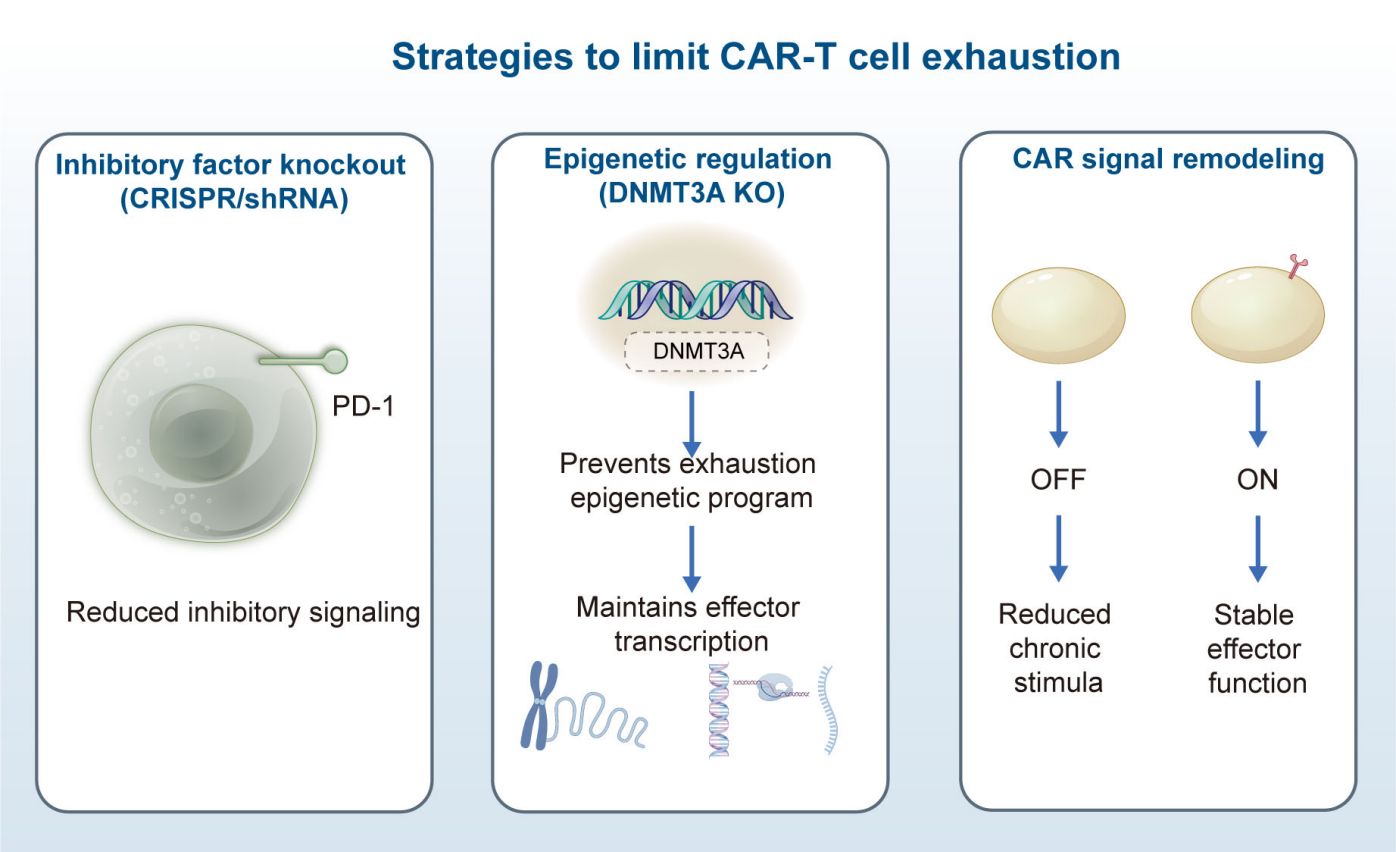
Strategies to limit chimeric antigen receptor (CAR) T-cell exhaustion. **(a)** Inhibitory Factor Knockout - Utilizing short hairpin RNA (shRNA) technology to target inhibitory receptors (e.g., PD-1), downregulating their cell surface expression levels; **(b)** Epigenetic Regulation - Knocking out the epigenetic regulator DNMT3A to block the accumulation of exhaustion-related epigenetic changes, maintaining gene transcriptional activity; **(c)** CAR Signal Remodeling - Designing a novel CAR structure that depends on the anchoring of auxiliary molecules. In the absence of this molecule, sustained signaling is blocked, preventing T-cell functional exhaustion induced by chronic stimulation.

CRISPR gene editing technology provides a powerful tool for developing exhaustion-resistant CAR-T cells (280). Through CRISPR editing, inhibitory receptor genes (e.g., PD-1 and CTLA-4) can be knocked out in CAR-T cells, shielding them from immunosuppression in the TME (**Figure 5**) (71, 281). CRISPR-edited CAR-T cells, with TCR, B2M, and PD-1 knocked out, generate universal CAR-T cells enhancing antitumor activity (282). Studies show that PD-1-knockout CAR-T cells exhibit approximately 3–5 times greater expansion capacity and a 60% increase in tumor clearance compared to unedited CAR-T cells in mouse leukemia models (283). Additionally, CRISPR editing enhances the metabolic adaptability of CAR-T cells. For example, knocking out the PTEN gene activates the PI3K-AKT signaling pathway, boosting glycolysis and oxidative phosphorylation activity in CAR-T cells, thereby improving their survival and function in the TME (284).

Currently, the efficacy of CAR-T in solid tumors is far inferior to that in hematological tumors. Can this situation be improved by shortening the *in vitro* culture time (from the traditional 2–3 weeks to less than 1 week)? (285, 286) This strategy has excellent clinical advantages: on the one hand, it can reduce the exhaustion/terminal differentiation of T cells during *in vitro* culture, possibly allowing for the return of more “young and vigorous” T cells; on the other hand, it will lower the manufacturing cost and increase accessibility. Moreover, the CRISPR/Cas9 system may pose risks of off-target cleavage and the introduction of immunogenicity, which can lead to unintended gene mutations and potential carcinogenicity (287). On this basis, more precise new-generation technologies such as high-fidelity Cas9 variants, optimized sgRNA design, base editing (Base Editing) or prime editing (Prime Editing) can be used to significantly reduce this risk. Moreover, the challenges of *in vivo* delivery and manufacturing still need to be addressed urgently. With the development of material science, the *in vivo* delivery of CRISPR components using liposomes to directly modify T cells may be able to improve this issue.

#### 4.3.2 Bispecific antibody-guided targeted activation of T cells

Bispecific antibodies (BsAbs) simultaneously bind to CD3 on the T-cell surface and specific antigens on the tumor cell surface, directing targeted activation of T cells to kill tumor cells (288). The first clinical validation of Blinatumomab (CD3×CD19 BsAb) demonstrated significant remission in B-cell malignancies (289). This targeted activation approach avoids the nonspecific toxicity that may occur in traditional CAR-T cell therapy. Studies show that BsAbs targeting CD3 and CD20 achieve a complete response rate of 80% in mouse lymphoma models, a 60% improvement compared to monotherapy with anti-CD20 (290). Research confirms the high antitumor activity of CD20×CD3 BsAbs in lymphoma models (291). BsAbs also regulate the intensity and duration of T-cell activation. By optimizing the affinity and structure of BsAbs, precise regulation of T-cell activation can be achieved. For example, in mouse colon cancer models, low-affinity BsAbs induce moderate T-cell activation, prolonging their survival *in vivo* and increasing tumor inhibition rates by approximately 40% (292). In clinical applications, BsAbs have shown promising therapeutic effects in various hematological and solid tumors. For instance, in metastatic breast cancer patients, BsAb treatment achieves an ORR of 35%, a 20% improvement compared to traditional chemotherapy (293). The targeted activation strategy of bispecific antibodies offers new perspectives for T-cell therapy with broad application prospects.

## 5 Conclusion

In summation, T-cell exhaustion plays a pivotal role in tumor immune evasion, wherein its intricate molecular mechanisms, dynamic plasticity, and interactions with the TME pose numerous challenges and opportunities for immunotherapy. A profound understanding of the differentiation pathways, functional attributes of exhausted T cells, and the mechanisms by which they are influenced by various elements of the TME is of paramount significance for precise diagnosis and treatment of tumors. Future research should concentrate on elucidating additional potential exhaustion-related biomarkers, developing novel drugs and therapeutic strategies capable of effectively reversing T-cell exhaustion, and simultaneously optimizing existing immunotherapy modalities such as combined application of epigenetic drugs, precise metabolic interventions, and engineered cell therapies. The overarching aim is to markedly enhance therapeutic efficacy and quality of life for cancer patients in clinical practice, thereby propelling tumor immunotherapy to new heights.
